# Studies on Counter Electrode Materials in Aqueous Zinc Half‐Cell Configurations

**DOI:** 10.1002/advs.77005

**Published:** 2026-08-07

**Authors:** Peiming Chen, Ruwei Chen, Xuhuan Xiao, Zhe Cui, Guanjie He

**Affiliations:** ^1^ Department of Chemistry University College London London UK

**Keywords:** current collector, electrochemistry, materials science, zinc

## Abstract

Coulombic Efficiency (CE) is the primary parameter that evaluates the reversibility of aqueous zinc‐ion batteries (ZIBs), as it directly reflects the stripping/plating behavior of zinc ions. However, this parameter is compromised by a lack of understanding and the standard of testing protocols. In the most ubiquitous method, half‐cell tests, the poor understanding of diverse metal substrates, namely the current collectors, compromises the reliability and comparability of the measured data. To timely fulfil this knowledge gap and establish a precise testing protocol, we selected four commonly used metal substrates, copper (Cu), titanium (Ti), stainless steel (SS), and aluminium (Al), to systematically compare their performance and disclose their underlying electrochemical reactions. The Ti current collector was determined to be the most reliable metal substrate due to its outstanding electrochemical performance with zinc ions and strong immunity to side reactions. Moreover, in this study, we found that most applied Cu current collectors exhibit severe side reactions and are likely to yield false‐positive results in environments with excessive zinc.

## Graphical abstract

Coulombic efficiency is a key metric for zinc reversibility in aqueous zinc‐ion batteries, but its reliability in zinc half‐cells is strongly influenced by poorly understood counter‐electrode materials. By systematically comparing Cu, Ti, stainless steel, and Al to identify reactions associated with current collectors, we find that Ti is the metal substrate with the highest zinc affinity and the fewest side reactions.

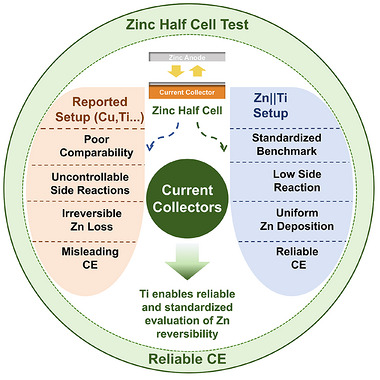

## 1 Introduction

Aqueous zinc ion batteries (AZIBs) are emerging as a potential ‘game changer’ in the modern energy storage landscape [1, 2, 3]. Although lithium‐ion batteries (LIBs) dominated the energy storage market for several decades, their intrinsic safety concerns make them unsuitable for large‐scale energy storage [4, 5]. In contrast, AZIBs offer significant advantages, including intrinsic safety, natural abundance, and a higher volumetric capacity of zinc anodes (5851 mAh/cm^3^ vs. 2046 mAh/cm^3^ for LIBs), rendering AZIBs highly attractive for large‐scale applications [6, 7, 8]. However, the practical deployment of AZIBs is severely impeded by challenges associated with the zinc anode. The uncontrolled dendrite formation, along with parasitic side reactions such as corrosion and the hydrogen evolution reaction (HER), results in poor reversibility [9, 10, 11, 12]. Various strategies have been devoted to addressing these issues through electrode modification, electrolyte optimization, electrolyte additives, and interface design [7, 13, 14, 15, 16]. The reported results appear remarkable, and more competitive works are being published. Despite the progress, a critical yet often overlooked aspect remains: the metal substrate (current collector), which serves as a fundamental component in coin cell configurations for AZIB evaluation, lacks an established standard and is not well understood [17, 18].

**FIGURE 1:**
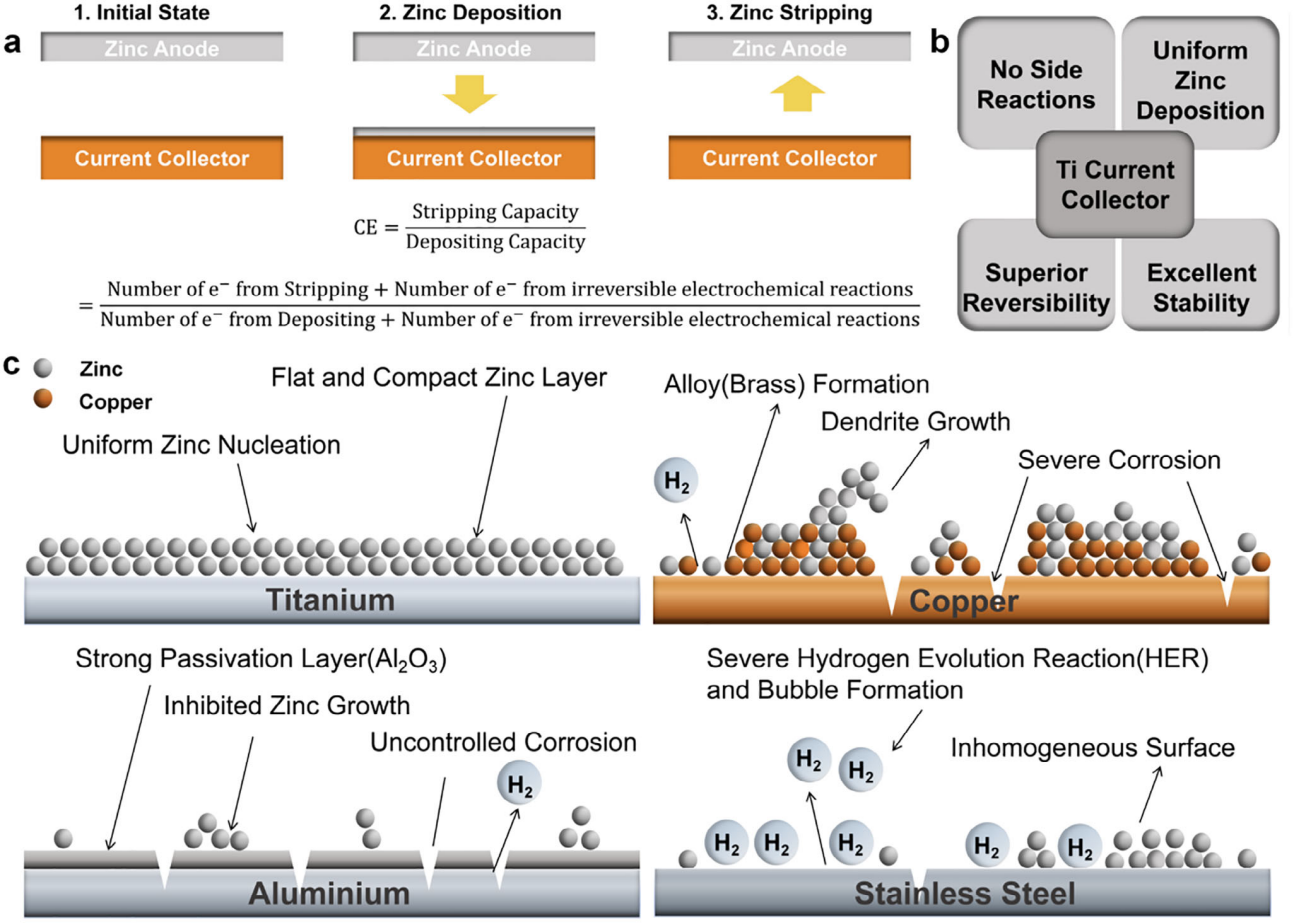
(a) Fundamentals of half‐cell test and Coulombic efficiency (CE); (b) Advantages of Titanium (Ti) current collector; (c) Interfacial behavior of Titanium, Copper, Aluminium, and Stainless Steel (SS) current collectors during half‐cell test.

Among all the reported works, Coulombic Efficiency (CE) is the most crucial parameter for quantifying the reversibility of the zinc stripping/plating process and assessing the effectiveness of the proposed strategies [17, 19, 20]. Theoretically, CE is defined as the ratio of the number of electrons associated with zinc deposition and subsequent stripping, which gives an intuitive way of assessing the zinc stripping/plating reversibility, as well as the presence of side reactions (Figure 1a) [20]. The most widely adopted method for determining CE is the asymmetric half‐cell test, first proposed by Xu et al. [1]. In this protocol, a Zn||metal substrate (copper) coin cell was used, with the metal substrate serving as the working electrode and zinc as the counter electrode. The CE is then calculated as the ratio of stripping to plating capacity. By eliminating interference from cathode redox processes, this approach provides a clear measure of zinc reversibility at the substrate interface. Over the past decade, this testing protocol has been extensively employed and has greatly facilitated progress in the field. However, because the calculated CE inherently reflects zinc reversibility on the selected metal substrate, the choice of substrate significantly affects the results, thereby influencing their accuracy and validity [21, 22]. Commonly used materials include copper (Cu), titanium (Ti), and stainless steel (SS). Cu, a widely adopted current collector in lithium half‐cell testing, was extended to AZIB research and remains the most popular option [23, 24]. Nevertheless, its stability in aqueous systems is questionable. Studies on current collectors for lithium batteries have shown that copper can undergo parasitic side reactions in the presence of residual water, raising concerns about its suitability for aqueous electrolytes [25]. Despite these concerns, Cu remains the dominant metal substrate in recent studies. For instance, zinc anode modification works published by the Liang and Mai groups both employed Zn||Cu configurations [26, 27]. In addition, an influential study on electrolyte additives from Guo's group also used this setup [28]. Ti is also seen as a viable candidate. Fan's work employed this metal substrate for anode modification, while Zhi's group employed Ti in hydrogel electrolyte systems [29, 30]. SS, although less commonly used, has also been tested, as demonstrated by Tang's group [31]. Recently published studies in AZIBs have also employed carbon‐based materials or carbon‐modified metal foils as anode current collectors [32, 33]. These substrates typically exhibit porous or 3D architectures, which differ substantially from the planar metal substrates commonly used in zinc half‐cell CE measurements. Therefore, the debate of the metal substrate material has been ongoing for a long time, and there still lack guidelines for material selection. The absence of established standards may undermine the reliability and comparability of CE measurements across the fields and pose a significant barrier to the development and validation of new strategies. Thus, systematic investigation and established standards for current collector materials are necessary.

To timely fulfil this knowledge gap and identify the most suitable material, we proposed a comparative study of four representative metal current collectors: Cu, Ti, SS, and aluminium (Al). Following the widely adopted testing protocol proposed by Xu [1], we systematically evaluated the influence of current collector species on CE measurement and plating/stripping behaviors. The coin cells were assembled with 2 m ZnSO_4_ electrolyte (150 µL) and a GF/D glass fibre separator. Moreover, to achieve a more realistic utilization rate, an ultrathin zinc foil (10 µm) was employed as the anode [34]. To understand the impact of metal substrates on reversibility and Zn plating/stripping behavior, electrochemical measurements, including galvanostatic charging/discharging (GCD), linear sweep voltammetry (LSV), and cyclic voltammetry (CV), were performed [35]. To further examine substrate‐induced side reactions and their influence on Zn stripping/plating behavior, material characterization techniques, including X‐ray diffraction (XRD) and scanning electron microscopy (SEM), were used. The results reveal that Ti performs best among the four representative metal current collectors. Notably, this superiority was maintained across three commonly used electrolytes with distinct properties. Systematic analysis suggests that the exceptional performance of Ti originates from its high resistance to side reactions, while simultaneously supporting a uniform and reversible zinc stripping/plating process (Figure 1b). These findings contrast with the conventional reliance on copper substrates and propose an important standard for AZIB testing. Overall, this work not only establishes Ti as a promising standard current collector for zinc half‐cell testing but also underscores the need for standardized protocols to ensure accuracy, precision, and reproducibility in evaluating zinc anodes (Figure 1c). This advancement will contribute to more reliable data and facilitate the rational design of next‐generation aqueous zinc‐ion batteries.

## 2 Results and Discussion

**FIGURE 2:**
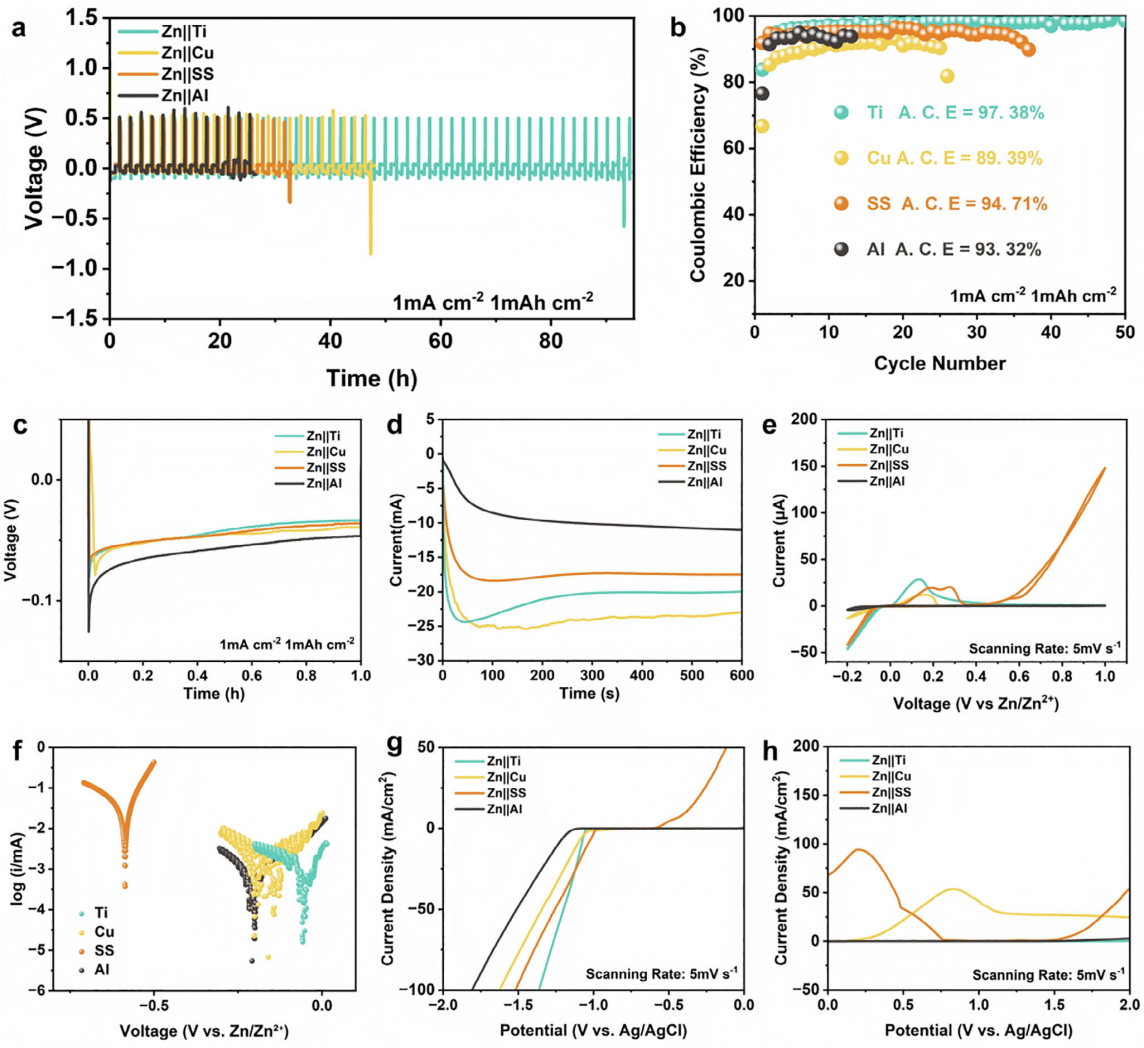
Electrochemical performance of Titanium, Copper, Stainless Steel, and Aluminium metal substrates with zinc foil (10 µm) in 2 m ZnSO_4_ (a) Galvanic Charge/Discharge Curve at 1 mA cm^−2^, 1 mAh cm^−2^ (b) According Coulombic Efficiency at 1 mA cm^−2^, 1 mAh cm^−2^ (c) Zinc nucleation overpotential (d) Chronoamperometry curves (e) Cyclic Voltammetry between ‐0.2–1.0 V, at the scanning rate of 5 mV/s (f) Tafel extrapolation fitting curves (g) Linear Sweep Voltammetry between ‐2.0–0 V with Ag/AgCl reference electrode (h) Linear Sweep Voltammetry between 0–2.0 V with Ag/AgCl reference electrode.

The choice of current collectors is critical for laboratory evaluation of proposed strategies in AZIBs. However, the lack of systematic understanding and standardized testing protocol challenges both ongoing and future studies. An ideal current collector should accurately reflect zinc plating/stripping behavior while remaining free from any intrinsic side reactions. To address this, we comparatively investigated four conventional current collectors widely used today: Cu, Ti, Al, and SS. Asymmetric half‐cells were assembled with 2 m ZnSO_4_ aqueous electrolyte (150 µL) and a glass fibre separator. Different current collectors served as cathodes to evaluate their effect on zinc plating/stripping behavior. To achieve a realistic zinc utilization rate comparable to industry and eliminate the influence of excessive zinc, we used ultrathin zinc foils (10 µm) as the zinc anode [34]. With this choice of extreme zinc foil thickness, the effect of excessive zinc supply is eliminated, and the CE measurement is more sensitive to zinc loss and side reactions induced by current collectors. To evaluate the plating/stripping behavior on different substrates, galvanostatic charge–discharge (GCD) cycling was performed. A current density of 1 mA/cm^2^ and an areal capacity of 1 mAh/cm^2^ were applied, with an upper cutoff voltage of 0.5 V. Notably, the cutoff voltage was adjusted to 0.45 V for the SS current collector to ensure reliable cycling behavior due to unstable SS above this potential. As shown in Figure 2a, Ti exhibits the longest cycle life, sustaining 85 h of stable operation under limited zinc supply. Cu shows a moderate cycle life of 40 h, outperforming SS (30 h) and Al (20 h). In addition to cycling stability, Coulombic efficiency (CE) is a key metric of electrochemical reversibility [1, 19, 20]. Compared to SS (94.71%), Al (93.32%), and Cu (89.39%), Ti shows the highest average CE of 97.38% (Figure 2b). It's worth noting that the CE and stability of Cu are not outstanding among all current collectors. This observation contradicts the idea that Cu is the most widely adopted current collector in published works [17, 21]. This discrepancy suggests that Cu may undergo complex side reactions that distort CE measurements. Thus, further investigation is necessary to analyze the zinc plating/stripping behavior and side reactions on different current collectors.

Nucleation overpotential is another crucial indicator of current collector performance [36]. The nucleation overpotential reflects the substrate's affinity for Zn^2^^+^ deposition, with lower values generally indicating a greater propensity for zinc deposition. As shown in Figure 2c, Ti exhibits the lowest nucleation overpotential, indicating a high affinity for zinc deposition. SS and Cu show slightly higher overpotentials, while Al displays the highest overpotential among the four current collectors. This implies that Al exhibits strong passivation toward zinc deposition, leading to suppressed zinc stripping/plating, shorter cycle time, and lower CE in Zn||Al cells. To further investigate zinc deposition behavior on various metal substrates, chronoamperometry (CA) measurements were performed under the same conditions. The curve in Figure 2d shows that Ti exhibits a rapid current response and a stable deposition current, indicating a strong affinity for zinc deposition and continuous zinc growth, consistent with the former analysis of the nucleation overpotential. In contrast, Cu current collectors exhibit a distinct CA profile compared to those of the other substrates. This variation may result from differences in zinc deposition behavior and the formation of zinc‐copper alloys. Additionally, the measurements for SS and Al are consistent with the previous results. The calculated Zn adsorption energies of various current collector materials further support this conclusion (Figure S2). Titanium exhibits the strongest zinc adsorption among all four current collectors, and the remaining calculation results are consistent with the measured trend.

During the asymmetric cell test, metal substrates are subjected not only to zinc deposition and stripping but also to competing side reactions [19]. To probe these processes more deeply, Cyclic voltammetry (CV) was performed using 2 m ZnSO_4_ electrolyte to ensure consistency with prior tests, with a scan rate of 5 mV s^−1^ to reflect the reactions observed during half‐cell tests. Figure 2e shows the CV performance of four current collectors over the selected range (‐0.2–1.0 V). The cathodic region (‐0.2 to 0 V) shows the same conclusion as the analysis of the nucleation potential: Ti and SS exhibit the best deposition behavior for Zn^2+^. In contrast, Cu and Al exhibit a weaker tendency to deposit zinc. On the anodic side, the SS current collector shows strong evidence of side reactions, with a drastic change in current, while other current collectors remain stable in this region. As mentioned previously, in the Zn||metal substrate half cells, the tested CE value is not only determined by zinc plating/stripping behavior but is also influenced by competing side reactions, such as corrosion, hydrogen evolution, and alloy formation. Thus, a compromised CE value indicates the intensity of side reactions and should be analyzed alongside other electrochemical performance tests to identify each parasitic reaction.

The corrosion behavior of current collectors was studied in a three‐electrode configuration using 2 m ZnSO_4_ aqueous electrolyte, with the tested metal as the working electrode, Ag/AgCl (3 m KCl) as the reference electrode, and Pt as the counter electrode. Tafel extrapolation was used to map the corrosion behavior (Figure 2f). Stainless steel shows a low polarization resistance and a rapid corrosion rate. In contrast, the others have relatively high resistance and a slower corrosion rate. However, during the experiment, the copper foil shows rapid degradation and fragmentation (Figure S1). Titanium has the highest polarization resistance among the tested metals. To further investigate the side reactions, linear sweep voltammetry (LSV) was performed. The cathodic performance of the current collector within the potential window of ‐2.0–0 V (Figure 2g) shows the same zinc deposition behavior as observed in the former tests. Stainless steel shows an abrupt increase between ‐0.6 and 0 V, indicating that the SS metal substrate undergoes a competitive reaction in this region. Anodic LSV curves (Figure 2h) show that the stainless steel current collector exhibits two regions, attributed to side reactions: the oxidation of the composition and the oxygen evolution reaction (OER). Meanwhile, the Cu current collector also shows side reactions throughout the anodic reaction window. Both Ti and Al show no evidence of side reactions in this stage.

**FIGURE 3:**
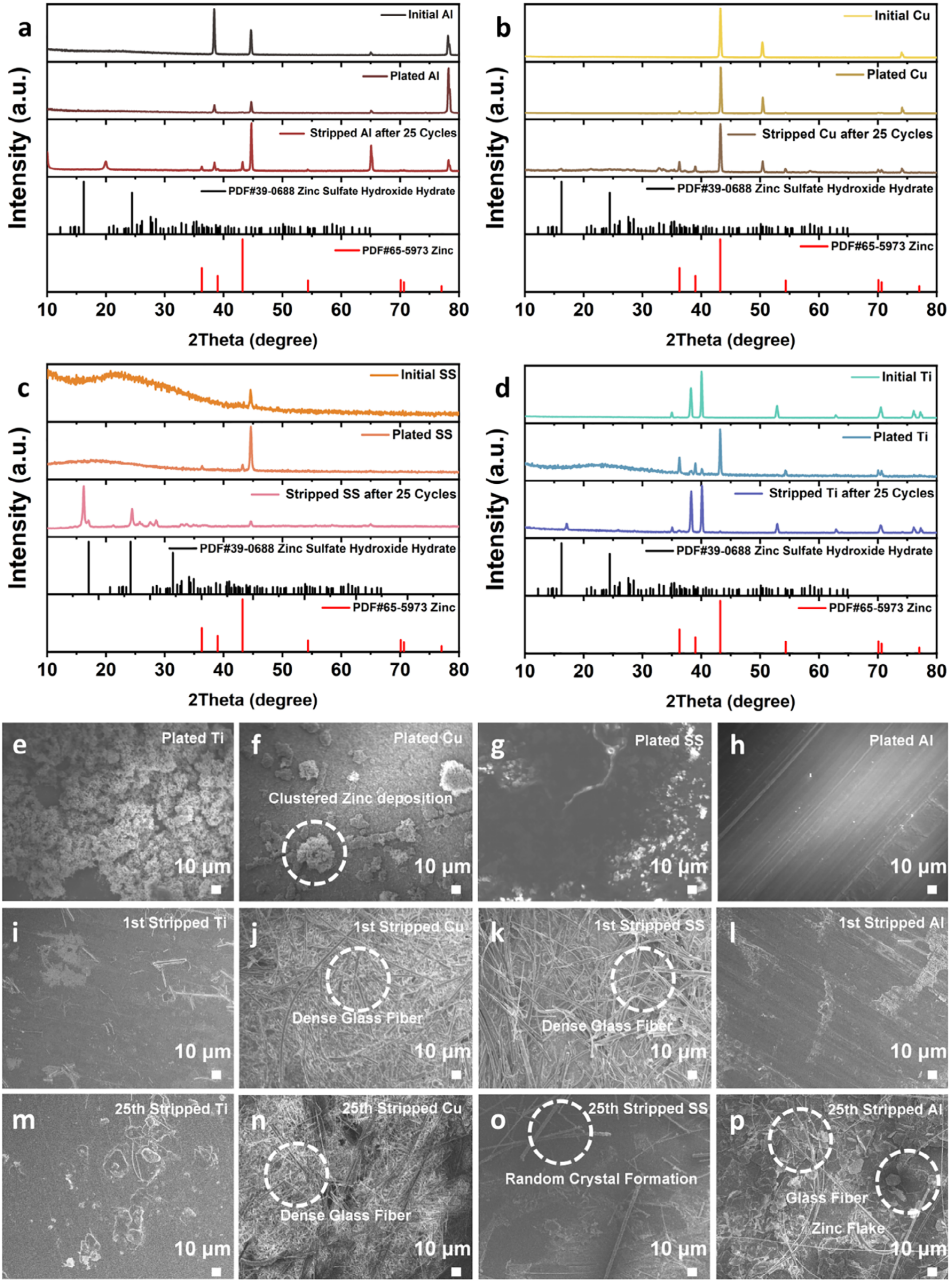
(a)‐(d) X‐ray Diffraction pattern of metal substrates after plating/ 25th stripping. (e)‐(h) Scanning Electron Microscopy image of metal substrates after plating; (i)‐(l) SEM images of metal substrates after first stripping (m)‐(p) metal substrates after 25th stripping.

In addition to the electrochemical performance of each current collector, the factors that determine the performance of the metal substrate are numerous. Zinc deposition behavior still needs to be investigated to examine its amount and morphology. During battery performance, the stripping of the zinc is also an essential indicator of the material property; if there is too much ‘dead zinc’ on the material surface, the half‐cell test will suffer from low CE, which is not associated with the zinc anode [19, 37]. Thus, the determination of residual zinc is crucial. Moreover, the byproduct species is the key factor in mapping the side reaction induced. X‐ray Diffraction (XRD) is an intuitive tool for identifying and quantifying crystalline materials [38]. The collected material is selected as the control group. In contrast, we selected the plated and stripped samples from the 25th cycle to investigate zinc deposition and residual zinc, along with the side reactions. Figure 3a–d demonstrates the XRD patterns of the selected material. Titanium, copper, and stainless‐steel current collectors all show patterns that align with the standard zinc peaks, while the zinc peaks are more intense for the titanium collector. On the other hand, the aluminium signal shows no evidence of zinc deposition, indicating strong passivation from the matte layer on the current collector surface [39]. Apart from the well‐deposited zinc, titanium also shows no evidence of residual zinc and byproducts. The copper current collector, on the other hand, shows not only the ‘dead zinc’ attached to the surface but also the characteristic peaks from two phases of brass, the alloy of zinc and copper. The 25th stripped sample shows a peak shift between 43° and 45°, corresponding to the shift of the copper (1 1 1) peak toward the (1 1 1) peak of α‐brass (Figure S3a) [40]. The peak around 50 ° indicates the formation of α‐brass as well. The 58° peak confirms the formation of β‐brass. The coexistence of two peaks implies that this reaction is spontaneous. Additionally, time‐dependent XRD patterns of the Cu current collectors were collected after the first, 10th, and 25th stripping cycles to clarify the evolution of brass formation (Figure S3b). The brass‐related diffraction signals become more evident with prolonged cycling, indicating that Zn/Cu alloying progressively develops during the Zn plating/stripping process. Therefore, brass formation is not a terminal product formed only after cell failure, but a cycling‐induced interfacial side reaction associated with the Cu current collector. This observation is also consistent with a previous study on electrodeposited Zn‐based anodes for aqueous zinc batteries, where Cu/Zn brass alloy phases were confirmed by XRD [41]. Stainless steel peaks exhibit a sharp reduction from the initial sample, which may be attributed to the dissolution of metal oxides in stainless steel. The lack of visible zinc peaks on the stripped sample indicates low ‘dead zinc’ formation; however, the high zinc sulfate hydrate peak intensity reflects the severe HER induced by this current collector. The aluminium crystal structure undergoes a drastic change during the test, indicating poor stability as a base material [39]. Moreover, the residual zinc is significant in the stripped sample at the 25th cycle. This phenomenon occurs even without excessive amounts of zinc; this behavior proves that the Zn||Al setup should be avoided in cell tests.

The morphology of zinc and the material surface is determined by scanning electron microscopy (SEM). The plated morphology is shown in Figure 3e–h. The aluminium shows no evidence of zinc deposition. At the same time, the copper current collector exhibits a cluster‐like deposition of zinc, which is prone to the formation of zinc dendrites [38]. The zinc deposition on SS and Ti both shows even and rigid morphology. We also conducted SEM characterization of stripped current collectors to deepen our understanding of side reactions, and we studied the morphology of the first and 25th cycles. The surface of the first stripped aluminium is as predicted, with the surface remaining clean, as there is no deposition during the first cycle. Meanwhile, the morphology of the 25th stripping is surprising. The residual zinc that cannot rejoin the reactions is in large amounts, while the glass fibre‐attached surface and random crystal growth both indicate the presence of side reactions [42]. Residual glass fibre contributes to the rough and irregular surface morphology, as its formation is caused by the trapping of glass fibre separators during cycling. The aluminium surface suffers severe corrosion, as current collection shows multiple hole regions. As shown in Figure 3k,o, the stainless steel surface from the first stripping cycle is covered with attached glass fibre, indicating irregular crystal formation [42]. However, the 25th stripped sample shows no unremovable glass fiber residues, and branch‐like ZHS crystals are visible. The reason for the glass fibre is not the formation of ZHS; it is suspected that the composition of the stainless steel's matte layer is oxidized during the stripping process, thus inducing the growth of irregular crystals [43]. With this possible change in composition, there is still no significant evidence of corrosion of the base material. The images of the stripped copper current collectors in Figure 3j,n show both to be covered with a dense glass fibre layer that is not removable by rinsing. Based on the analysis of the stripped SS, it is questionable whether the attachment of the glass fibre is due to the ZHS. As illustrated previously, the formation of brass is a spontaneous process; thus, the reason for the glass fibre attachment is the brass formation, which causes a coarsened surface. Moreover, the dimpled surface indicates corrosion of the metal substrate. Both stripped titanium surfaces are intact and covered with glass fibre, indicating a smooth surface. The surface of titanium does not exhibit corrosion during charging and discharging, and there is no valid evidence of residual zinc, nor is ZHS crystal growth severe. Both the stripping and plating processes demonstrate that titanium exhibits excellent anti‐corrosion properties and also possesses outstanding zinc plating and stripping performance. Overall, SS and Al are also ruled out as selection criteria for metal substrates for the CE test of AZIBs. SS is limited by significant side reactions, and its unstable surface reactions reduce its reliability in half‐cell tests. Al exhibits minimal zinc deposition, and its passivation is accompanied by unavoidable corrosion. Therefore, SS and Al are not recommended as current collectors for AZIB testing.

**FIGURE 4:**
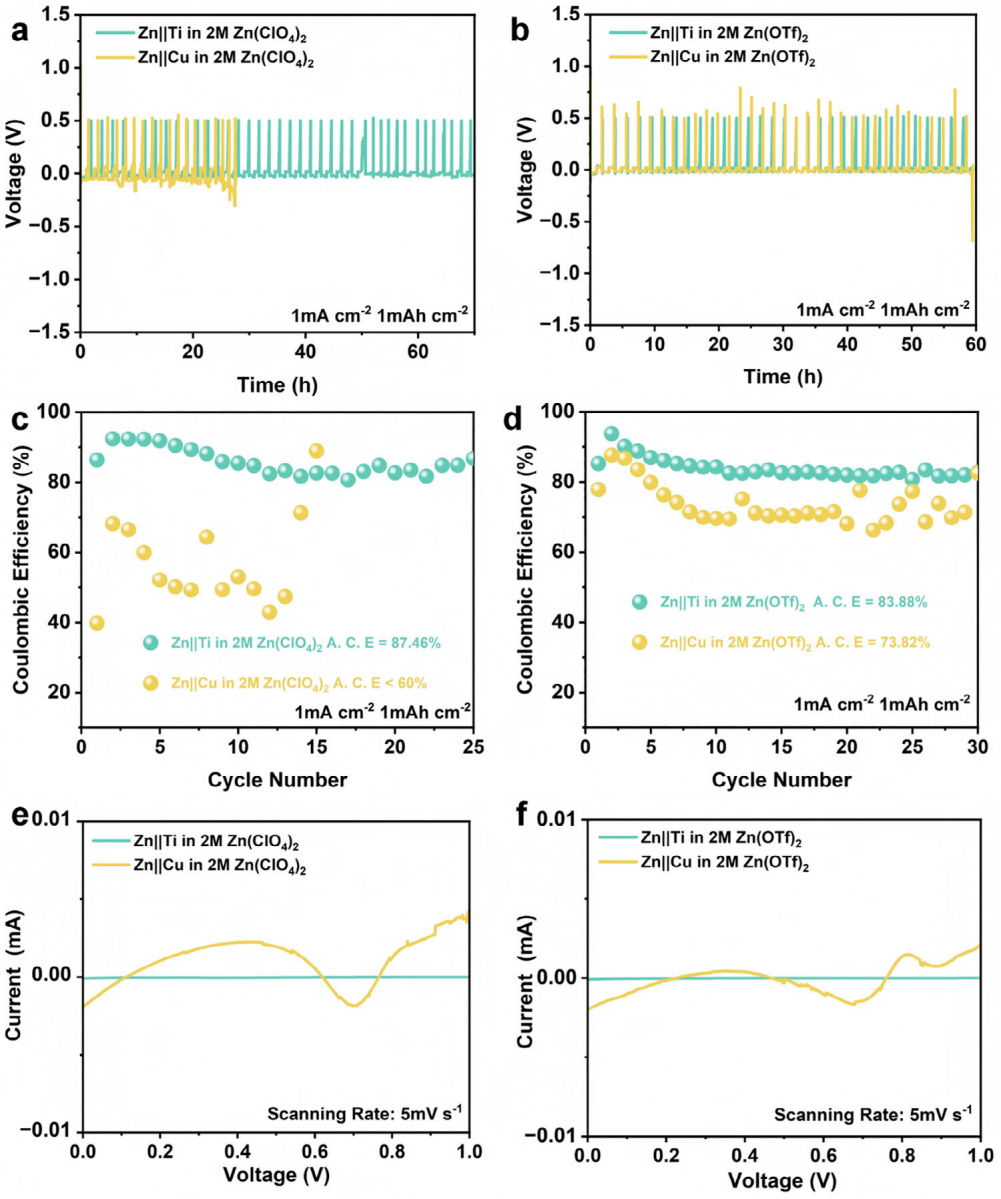
Electrochemical performance of Titanium and Copper (a,b) Galvanic Charge/Discharge Curve at 1 mA cm^−2^, 1mAh cm^−2^ in 2 m Zn(ClO_4_)_2_ electrolyte and 2 m Zn(OTf)_2_ electrolyte (c,d) Coulombic Efficiency at 1 mA cm^−2^, 1mAh cm^−2^ in 2 m Zn(ClO_4_)_2_ electrolyte and 2 m Zn(OTf)_2_ electrolyte (e,f) Linear Sweep Voltammetry between 0–1 V with coin cell in 2 m Zn(ClO_4_)_2_ electrolyte and 2 m Zn(OTf)_2_ electrolyte.

In the previous part, titanium demonstrated superior performance compared to other candidate materials. However, all the experiments were conducted using 2 m ZnSO_4_ as the electrolyte. Despite zinc sulfate being the dominant AZIB electrolyte and representative of a neutral‐to‐mildly acidic electrolyte environment, the types of zinc electrolyte systems studied today remain numerous. To further prove the advantage of applying titanium as the current collector material in different electrolyte systems for AZIBs, we selected zinc perchlorate (Zn(ClO_4_)_2_) and zinc triflate (Zn(OTf)_2_) to be the alternative electrolytes. The comparison is set with the conventional copper current collector, as it is the most reported half‐cell metal substrate for alternative electrolytes. Zn(ClO_4_)_2_ is a commonly used corrosive zinc electrolyte for AZIBs [44]. It is also notorious for its high oxidizability, as shown in Figure 4a, where the copper current collector exhibits minimal performance during the test. In contrast, the titanium current collector demonstrates significantly higher performance. The CE shown in Figure 4c indicates the same conclusion; the titanium current collector A.C.E. is 87.46%. This again proves the anticorrosion properties of titanium. Zinc triflate is another common zinc electrolyte. It exhibits lower hydration effects, indicating a reduced tendency for water‐induced corrosion and improved performance [1, 19]. However, this electrolyte is unrealistic for industrial use due to its high price [45]. The test results in Figure 4b,d show similar cycle life for titanium and copper current collectors, and the A.C.E. of the two current collectors approaches each other. The anodic behavior of Ti and Cu current collectors was investigated using LSV in coin cells (Figure 4e,f). In both electrolytes, Cu is more vulnerable to side reactions, while Ti remains stable in different electrolyte environments. Additional CA, CV, cathodic LSV, and Tafel analyses are presented in Figures S4–S7. Collectively, these electrochemical results demonstrate that the Ti current collector possesses superior chemical stability.

**FIGURE 5:**
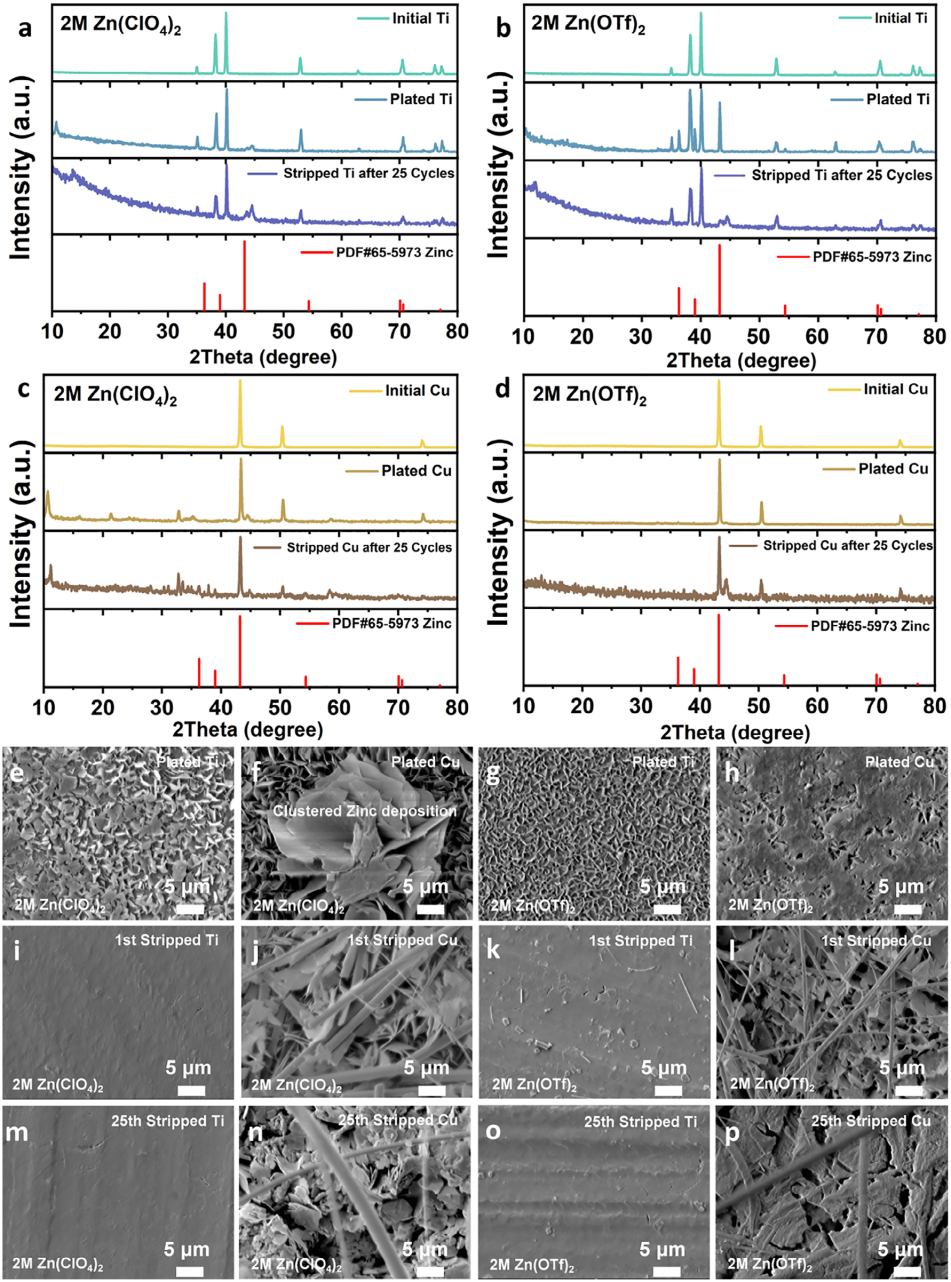
(a)‐(d) X‐ray Diffraction pattern of Titanium and Copper substrates, after plating/ 25th stripping in 2 m Zn(ClO_4_)_2_ electrolyte and 2 m Zn(OTf)_2_ electrolyte (e)‐(h) Scanning Electron Microscopy image of metal substrates after plating (i)‐(l) SEM images of Ti and Cu substrates after first stripping (m)‐(p) Ti and Cu substrates after 25th stripping in 2 m Zn(ClO_4_)_2_ electrolyte and 2 m Zn(OTf)_2_ electrolyte.

To further investigate the electrochemical behavior of Ti and Cu in various electrolyte environments, XRD and SEM characterisations were conducted for plated/stripped Ti and Cu current collectors to observe their interfacial morphology. As shown in Figure 5a–d, the Ti metal substrate exhibits distinct zinc deposition peaks and less pronounced by‐product formation peaks. Additionally, the stripped XRD measurement of Ti demonstrates superior reversibility of zinc plating and stripping. In contrast, the Cu current collector performs worse than the ZnSO_4_ electrolyte; for both electrolytes, the zinc deposition peaks are weak, while by‐product formation is more pronounced, as indicated by the sharp peaks in the measurements.

SEM images further support these findings. Figure 5e–h illustrate uniform and smooth zinc plating on the Ti substrate, whereas zinc deposition on Cu appears clustered and highly irregular. The first‐cycle stripping morphology (Figure 5i–l) reveals that the Ti surface remains intact and clean after deposition, while the Cu surface is covered with dense glass fiber and residual zinc.SEM images after the 25th stripping cycle (Figure 5m–p) indicate that Ti is more corrosion‐resistant, whereas Cu exhibits a deteriorated morphology, including corrosion pits and increased residual zinc. These material characterization results align with those in Figure 4 and confirm that Ti demonstrates more reliable interfacial behavior than Cu across different electrolyte systems.

## 3 Conclusion

This work reflects on the existing testing protocol for Zn CE, which revealed a lack of understanding regarding current collector materials, leading to unregulated testing schemes. At the same time, the unknown mechanism of zinc deposition and the side reactions hinder the precision of the testing results. Compared with conventional current collectors, the titanium current collector is the most promising metal substrate for zinc half‐cell tests. The Zn||Ti setup can ensure accurate measurements due to its high corrosion resistance, stable zinc plating/stripping performance, and immunity to external side reactions. On the other hand, the widely applied copper current collector shows surprisingly poor electrochemical properties. The copper current collector is vulnerable to corrosion in aqueous electrolyte, and nonuniform zinc deposition can lead to misjudgment of zinc anode performance. The mass forming of zincophilic brass might be the reason for the high performance of Zn||Cu under the excessive use of zinc in published works. Thus, we suggest eliminating the use of copper material in half‐cell tests. Moreover, we explained why stainless steel and aluminium are seldom used in half‐cell tests. The SS exhibits good zinc stripping and plating performance; however, its performance is hindered by the dissolution of its own matte layer and side reactions with the electrolyte. On the other hand, the aluminium current collector's zinc deposition is hindered by the dense matte layer, while the crystal structure of aluminium remains unstable. The corrosion of aluminium in a water‐based electrolyte is severe, making it the least desirable current collector. The establishment of this setup standard will have a substantial impact toward the AZIBs studies. With this new testing protocol, future studies will be more systematic and easier to compare (Figure S8). Studies of the current collector mechanism will also provide insight into other aqueous‐based battery systems. In the future, we should prevent a similar illusion, such as the ‘false good performance’ seen with brass.

## Author Contributions

**Guanjie He** conceptualized and supervised the research. **Peiming Chen** conducted the experiment and wrote the first draft. Peiming Chen and **Xuhuan Xiao** performed characterisation. Peiming Chen, Xuhuan Xiao, and **Zhe Cui** designed the graphs. **Ruwei Chen** and Guanjie He revised the paper.

## Conflicts of Interest

The authors declare no conflicts of interest.

## Supporting information




**Supporting File**: advs77005‐sup‐0001‐SuppMat.docx.

## Data Availability

The data that support the findings of this study are available from the corresponding author upon reasonable request.
