# Supplementary material for: Studies on Counter Electrode Materials in Aqueous Zinc Half‐Cell Configurations
Source: Adv Sci (Weinh). 2026 Aug 7:e77005. Online ahead of print. doi: 10.1002/advs.77005 (PMC13449537; doi:10.1002/advs.77005)
Supplement: Supplementary file 1 [file ADVS-9999-e77005-s001.docx]

**Supporting Information**

**Experimental section:**

1. **Characterisations**

X-ray diffraction(XRD) was conducted from Panalytical Empyrean diffractometer with Cu Kα radiation. The surface morphologies were obtained from JEOL 7600, all samples were coated with gold prior to measurement.

2. **Electrochemical measurements**

The coin cells were assembled with CR2032 coin cell cases in open air environment, using 2M ZnSO_4_, Zn(ClO_4)2_, and Zn(OTf)_2_ as electrolyte (150 μl), with glass fiber separator (Whatman, GF/D), zinc anode (commerical zinc foil, 10μm), and metal substrate as cathode (commercial aluminium, copper, titanium, stainless steel foil). All metal foils were ultrasonically cleaned in deionised water in 30 minutes, then repeat the same process in ethanol. The coin cells were GCD tested with Neware electrochemistry workstation in 28 °C. CV tests, EIS tests, and LSV tests for were performed with Biologic VSP workstation. For tests with H-cell, the electrode assembly was same as the previous setting, and electrolyte is 2M ZnSO_4_ (60mL). LSV tests and Tafel extrapolation were conducted with Gamry.

3. **Calculation of adsorption energy**

All first principle calculation (Ab initio) calculations were accomplished using Vienna Ab initio simulation package (VASP) [1, 2]. The generalized gradient approximation (GGA) functionals were considered by PerdewBurke-Ernzerh as well as projector augmented wave method (PAW-PBE) to describe exchange-correlation effects between electrons [3-5]. A 15.0-Å vacuum layer was introduced on the Z-axis direction to limit interactions between periodic images. The supercells with four-layer slabs were chosen to represent the adsorbed surface for molecules, and the bottom two layers were fixed to maintain the bulk property. For different substrate models, the k-point meshes were automatically adjusted according to the lattice dimensions while maintaining the same k-point spacing of 0.03 Å⁻¹. The energy cutoff for the plane-wave basis set was 520 eV. In the optimization process, the convergence criterion for the electronic self-consistent field (SCF) and loop was set to 1×10-6 eV/atom, in the single energy process the convergence criterion for the electronic self-consistent field (SCF) and loop was set to 1×10-7 eV/atom. All the structures are relaxed until the residual forces on the atoms have declined to less than 0.02 eV Å-1. For all calculations, Van der Waals interaction was taken into account at DFT-D4 [6]. The adsorption energy is calculated via:

$E_{ads}=E_{slab+mol}-E_{slab}-E_{mol}$(S1)

where the Eslab+mol, the Eslab, and the Emol represent the total energy of adsorbates on the Slab, the energy of the Slab, and the energy of the isolated adsorbates, respectively.

Figure S1. Photographs of Copper current collector after Tafel extrapolation

Figure S2. Calculated Zn adsorption energies on main elements of different current collector materials

Fig S3. (a) X-ray Diffraction pattern of Cu current collector after 25th stripping and (b) time-dependent XRD patterns of Cu current collectors after different stripping cycles

Figure S4. Chronoamperometry curves of Titanium and Copper in (a) 2 M Zn(ClO₄)₂ and (b)2 M Zn(OTf)₂ electrolytes

Figure S5. Cyclic Voltammetry curves of Titanium and Copper in (a) 2 M Zn(ClO₄)₂ and (b)2 M Zn(OTf)₂ electrolytes

Fig S6. Linear Sweep Voltammetry between -0.5V-0V with coin cell in 2M Zn(ClO_4_)_2_ electrolyte and 2M Zn(OTf)_2_ electrolyte

Figure S7. Tafel extrapolation fitting curves of Titanium and Copper in (a) 2 M Zn(ClO₄)₂ and (b)2 M Zn(OTf)₂ electrolytes

Figure S8. Multidimensional comparison of current collector performance

**References:**

[1] G. Kresse, J. Hafner, Ab initio molecular-dynamics simulation of the liquid-metal–amorphous-semiconductor transition in germanium. Phys. Rev. B 49, 14251–14269 (1994).

[2] G. Kresse, J. Furthmüller, Efficiency of ab-initio total energy calculations for metals and semiconductors using a plane-wave basis set. Comput. Mater. Sci. 6, 15–50 (1996).

[3] P. E. Blöchl, O. Jepsen, O. K. Andersen, Improved tetrahedron method for Brillouin-zone integrations. Phys. Rev. B 49, 16223–16233 (1994).

[4] G. Kresse, D. Joubert, From ultrasoft pseudopotentials to the projector augmented-wave method. Phys. Rev. B 59, 1758–1775 (1999).

[5] J. P. Perdew, K. Burke, M. Ernzerhof, Generalized Gradient Approximation Made Simple. Phys. Rev. Lett. 77, 3865–3868 (1996).

[6] M. Murbach, B. Gerwe, N. Dawson-Elli, L. Tsui, impedance.py: A Python package for electrochemical impedance analysis. JOSS 5, 2349 (2020).
